# Retraction: Recent global warming as a proximate cause of deforestation and forest degradation in northern Pakistan

**DOI:** 10.1371/journal.pone.0274410

**Published:** 2022-09-14

**Authors:** The PLOS ONE Editors

The *PLOS ONE* Editors retract this article [1] because it was identified as one of a series of submissions for which we have concerns about authorship, competing interests, and peer review. We regret that the issues were not addressed prior to the article’s publication.

SaifU, TG, SA, MMW, ANS, and SamiU did not agree with the retraction. NMS and RSN responded but expressed neither agreement nor disagreement with the editorial decision.
